# Integrating Biological Architecture and Biomaterial Function: Exploring the Native Hydrogel Structure of Brown Seaweed

**DOI:** 10.1002/mabi.202500622

**Published:** 2026-04-07

**Authors:** Linn Berglund, Richa Sharma

**Affiliations:** ^1^ Division of Materials Science, Department of Engineering Sciences and Mathematics Luleå University of Technology Luleå Sweden

**Keywords:** absorption, alginate, cytotoxicity, kelp, nanocellulose, structure

## Abstract

Brown seaweed is a naturally occurring composite that integrates alginate and cellulose within a hierarchical, hydrated architecture analogous to engineered hydrogel systems. This study hypothesizes that leveraging the native structure–function relationships of brown seaweed enables the development of functional hydrogel biomaterials while minimizing synthetic and chemical processing. Strategies are investigated to exploit the intrinsic biological structure and composition of brown seaweed blades across multiple formats, including native and purified blade structures, as well as fibrillated blades reassembled into hydrogels and foam structures via 3D printing and freeze‐drying. The resulting biomaterials are characterized in terms of structure, hydrogel stability, and liquid absorption capacity in different media. The effects of purification are compared with those of native materials. In addition, porosity, mechanical, rheological, and cytocompatibility properties of the fibrillated and reassembled structures are evaluated. By preserving the natural architecture and avoiding extensive fractionation, this approach demonstrates the potential to create resource‐efficient biomaterials with high liquid absorption (∼3600%), high porosity (∼93%), and shape‐memory behavior after compression. Cytocompatibility reaches ∼73% viability at 50% extract but decreases to ∼59% at full concentration, indicating a concentration‐dependent biological response, underscoring the need to balance minimal processing with biological performance for biomedical applications.

## Graphical abstract

This study explores the natural internal structure of brown seaweed and how it influences material behavior. By preserving and reassembling the seaweed's native architecture, hydrogels and foams with high liquid absorption, porosity, and shape recovery were created using minimal processing. The results highlight how biological structures can guide the design of sustainable biomaterials.

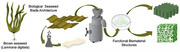

## 1 Introduction

In the pursuit of resource‐efficient and functional materials, brown seaweed represents a promising renewable alternative to traditional petroleum‐derived and resource‐intensive substances. Among the various species, *Laminaria digitata*—a fast‐growing, cold‐water brown macroalga—stands out due to its distinctive biochemical composition and structural characteristics. *Laminaria digitata*, commonly referred to as kelp, forms dense underwater forests along coastal regions. Its lamella‐structure grows in the upper sublittoral zone of rocky, wave‐exposed environments, and it is highly adapted to these conditions through pronounced mechanical flexibility. The cell wall architecture differs from that of terrestrial plants; although cellulose is present, alginate constitutes the dominant polysaccharide, forming a rigid yet flexible network that enables the organism to withstand hydrodynamic forces [1]. Rich in bioactive and functional compounds such as alginate, laminarin, mannitol, fucoidan, and cellulose fibers [2], *Laminaria digitata* offers significant potential for the development of multifunctional materials across a wide range of application areas.

The major natural polymer present in *Laminaria digitata*, alginate, provides a versatile foundation for material innovation. Alginate is a linear copolymer of β‐D‐mannuronic acid (M) and α‐L‐guluronic acid (G) monomer units, arranged in M‐blocks, G‐blocks, and MG‐blocks [3]. In addition to alginate, brown seaweeds contain other polysaccharides, including cellulose, laminarin, and fucoidan, which contribute to key material attributes such as biocompatibility, non‐toxicity, and natural bioactivity [4]. Consequently, seaweed represents a favorable and sustainable resource with broad potential in biomedical material development.

The cultivation of brown seaweed is characterized by rapid growth rates and the ability to thrive in marine environments without the need for freshwater, fertilizers, or arable land, making it an ideal candidate for circular material innovation [5]. As industries seek to decarbonize supply chains and reduce ecological footprints, harnessing the potential of *Laminaria digitata* represents a promising avenue for the development of sustainable materials that align with both environmental sustainability and economic innovation. This largely untapped marine biomass not only supports environmental objectives but also creates new economic opportunities within the blue economy, fostering resilience and innovation in response to global resource challenges.

As an anionic polymer, alginate enables material preparation under mild, aqueous, and ion‐mediated conditions [6]. The free carboxylate groups along the polymer backbone readily undergo ionic crosslinking with divalent or multivalent ions or molecules [7]. In addition, the presence of multiple functional groups, including primary and secondary hydroxyl groups and carboxylates, supports its versatility in applications such as wound dressings [8], tissue engineering, cell and probiotic encapsulation, and drug delivery systems [3]. Alginate‐based materials in the form of films, hydrogels, fibers, and sponges provide a moist wound environment, protect against external contamination, and absorb moderate to high levels of exudate depending on material form [9]. Alginate hydrogels and foams further contribute to inflammation control by maintaining moisture and managing exudate during the wound‐healing process, thereby helping to prevent infection. The mild and aqueous crosslinking conditions also enable the survival of encapsulated biological agents [10].

Despite these advantages and the demonstrated potential of alginate‐ and cellulose‐based materials for wound healing, drug delivery, and tissue engineering, existing studies predominantly focus on isolated or purified polymers [11, 12]. Such approaches rely on extensive extraction, purification, and fractionation steps that separate alginate and cellulose prior to material fabrication and incorporation into biomedical systems [13]. This strategy neglects the native hierarchical organization and compositional synergy present in intact seaweed tissue. Such processing often results in the loss of structural integrity, compositional synergy, and cost‐effectiveness of the raw biomass, and can adversely affect biological functionality. Consequently, current alginate or nanocellulose hydrogels do not exploit the intrinsic structural integration of these components as they occur in nature. The knowledge gap addressed in this work is therefore whether the naturally occurring alginate–cellulose composite architecture of brown seaweed can be directly leveraged as a functional hydrogel system, reducing processing intensity while maintaining or enhancing material performance.

In a previous study, it was demonstrated that cellulose and alginate present in *Laminaria digitata* can be isolated simultaneously to produce alginate‐cellulose nanofibers exhibiting favorable rheological behavior, cytocompatibility, and 3D‐printability [6]. Although approximately 23% of cellulose and 46% of alginate were retained, the process still involved chemical pretreatment, with an overall yield of about 70 wt.%, indicating that roughly 30 wt.% of the raw biomass was lost. Consequently, the development of processing routes that enable the direct utilization of seaweed biomass with minimal processing represents a more sustainable and efficient alternative for material development. Direct or minimally processed seaweed retains both cellulose and alginate as well as low‐abundance constituents such as fucoidan, phlorotannins, phenolics, minerals, pigments, and oligomers, which are often removed during chemical treatments. These bioactive components have been shown to exhibit antioxidant and anti‐inflammatory properties [14, 15], as well as antimicrobial activity [16], contributing to the development of functional materials.

The present work is described as a bioinspired design concept enabled by a minimal processing strategy. Brown seaweed itself is a naturally occurring composite; however, the novelty of this study lies in systematically leveraging its intrinsic hierarchical alginate–cellulose architecture to create functional hydrogel biomaterials while avoiding extensive fractionation or synthetic modification. This study hypothesizes that harnessing the native structure–function relationships of brown seaweed enables the development of functional hydrogel biomaterials while minimizing synthetic and chemical processing. The intrinsic biological structure of the brown seaweed blade is explored for the development of resource‐efficient biomaterial structures, characterized as hydrogels. Multiple strategies are applied to elucidate the potential of brown seaweed across different material formats, including native blade structures, purified blade structures, and fibrillated blades reassembled via 3D printing and freeze‐drying. The objective of this work is to evaluate whether minimally processed brown seaweed can be transformed into porous biomaterial architectures with defined mechanical properties, structural integrity, and high fluid absorption capacity. In parallel, the cytocompatibility of fibrillated seaweed gel is assessed to provide an initial indication of biological response, relevant to biomedical use. By examining how preservation of the native lamellar organization and intrinsic composition influences material performance and biological response, this study aims to elucidate strategies for balancing sustainability‐driven minimal processing with the functional requirements of biomedical systems.

## 2 Results and Discussion

**FIGURE 1:**
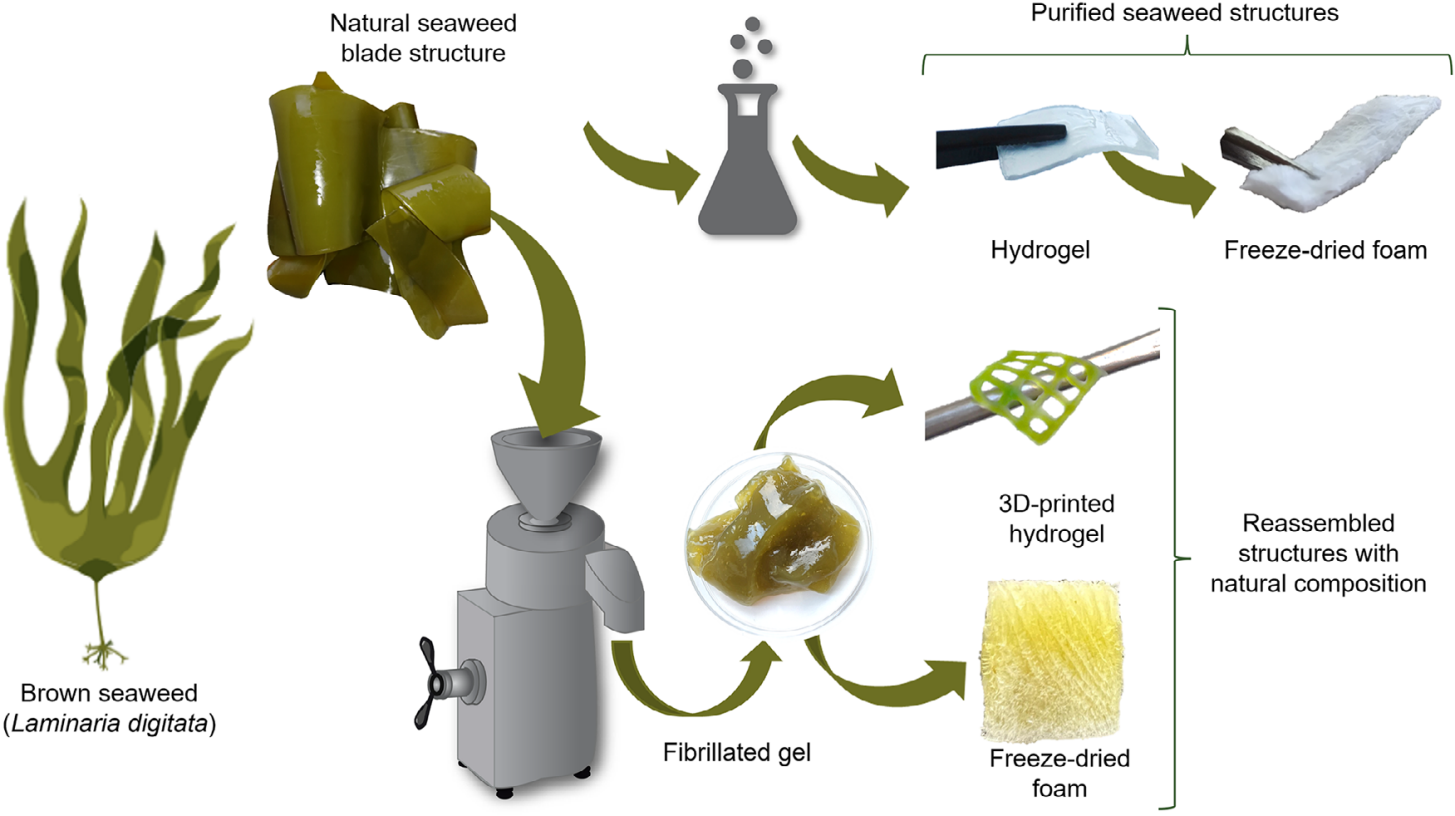
Overview of the processing routes for the developed biomaterial structures.

The blades of brown seaweed were processed into different material structures, including purified and reassembled forms, as illustrated in Figure 1, following distinct processing routes. In the first route, the blades were chemically pretreated to obtain a purified hydrogel, which was subsequently freeze‐dried to produce a freeze‐dried foam. In the second route, the blades were fibrillated at solid contents of 2 and 4 wt.% to obtain gels with the natural composition preserved, which were subsequently used as inks for 3D printing or freeze‐dried to generate freeze‐dried foams.

### 2.1 Natural Seaweed Blade Structures

**FIGURE 2:**
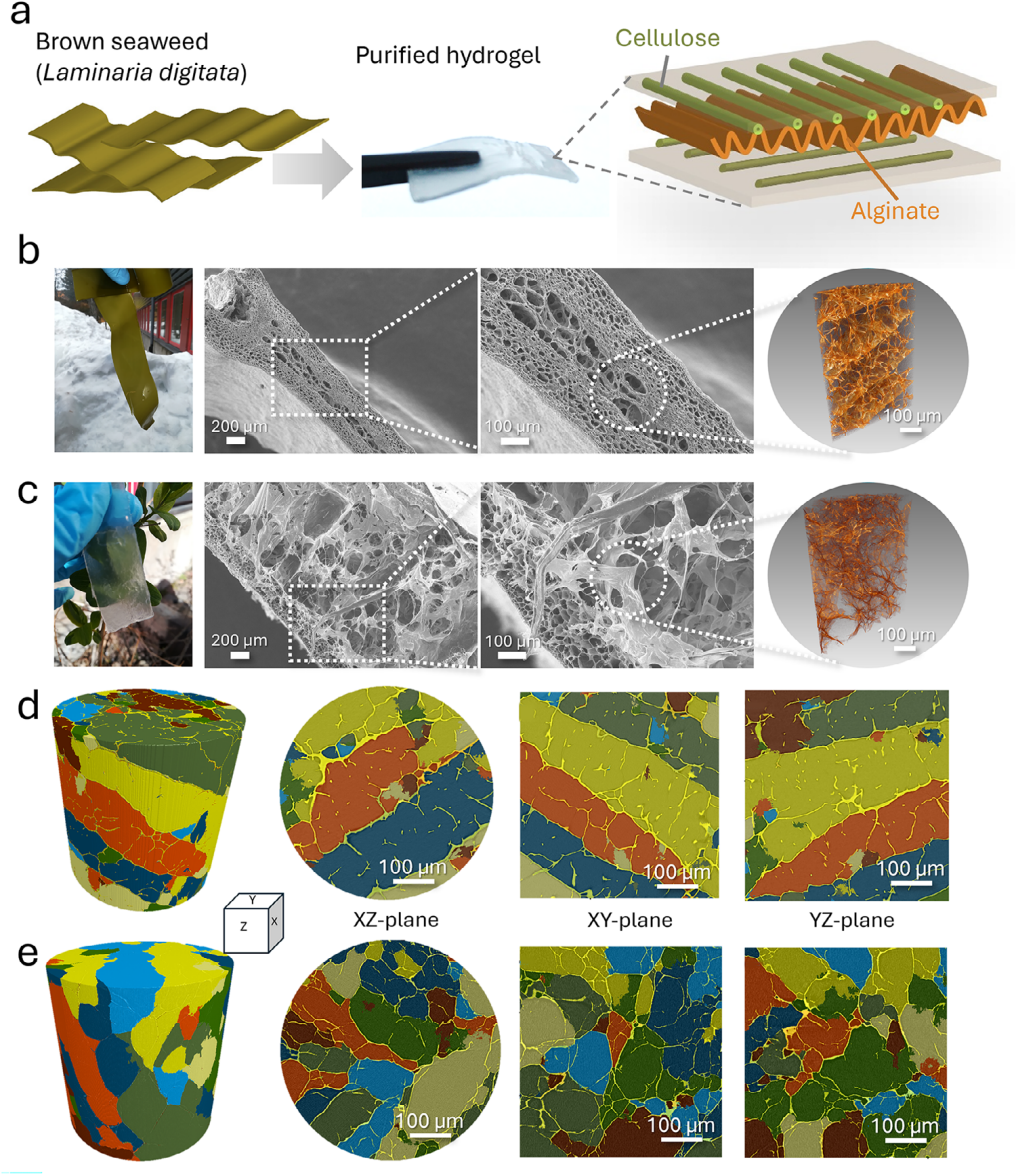
(a) Overview of the raw blade structure and the purified hydrogel structure with schematic visualization of the alginate and cellulose components preserved. Photos, SEM images of cross‐sections, and XRT 3D visualizations 20× objective, FOV 0.56 mm (from left to right) of (b) raw blade structure, and (c) purified hydrogel structure, respectively. Segmentation from XRT 3D visualizations of (d) raw blade structure, and (e) purified hydrogel structure for XZ‐, XY‐, and YZ‐planes (from left to right).

To better understand the native structure of seaweed blades, microscopic characterization was performed using scanning electron microscopy (SEM) to analyze the material's cross‐sections and X‐ray microtomography (XRT) to visualize the three‐dimensional (3D) internal architecture. Raw seaweed was characterized in its native state and after purification by sodium chlorite (NaClO_2_) bleaching. The effects of purification on seaweed structure were evaluated by comparison with non‐purified samples, as presented in Figure 2.

The objective of the seaweed purification process was to reduce pigments and other impurities such as chlorophyll, carotenoids, phenolic compounds, proteins, lipids, and mineral salts [2], while preserving the overall structural integrity of the material. Following sodium chlorite bleaching, the purified hydrogel structure remained visually intact, as shown in Figure 2a,c, although it appeared translucent in contrast to the greenish raw blade structure (Figure 2b, Figure S1). Previous work has demonstrated that both cellulose and alginate are largely preserved after the bleaching process [17]. SEM imaging of the raw blade (Figure 2b) revealed a layered architecture. Smaller pores dominated the outer layers of the cell wall structure, while larger pores were present in the central layers. XRT visualization of the internal structure provided detailed insight into this layered morphology, revealing elongated, in some regions, tubule‐like structures, with interconnected pores between layers (Figure 2b). These features were also evident in two‐dimensional XRT cross‐sectional images (Figure 2d, Figure S2). The observed architecture is consistent with previous transmission electron microscopy studies of *Laminaria digitata* ultrastructure [18]. After purification, the structure displayed a more open pore structure, particularly within the internal layers, as observed by SEM and XRT analyses (Figures 2c and e). Pore‐size distribution histograms derived from XRT reconstructions are presented in Figure S3, showing a comparable overall pore‐size range for both raw and purified samples (5–450 µm). The distributions indicated a higher frequency of smaller pores (≤40 µm) in the raw material compared to the purified structure, whereas pores of intermediate size (∼50 µm) occurred more frequently in the purified material (approximately 30%) than in the raw material (approximately 24%). These trends were also consistent with the qualitative observations from SEM images (Figure 2b,c). Notably, a low‐frequency occurrence of larger pores (200–400 µm) was observed in the raw structure but was not detected in the purified samples. This difference may be partially attributed to limitations in imaging resolution and low X‐ray contrast between the material and pore spaces in the raw structure (Figure 2d), which complicated the accurate identification of interconnected pores. These observations highlighted the need for further characterization to more fully elucidate the hierarchical pore architecture of seaweed‐based materials.

**FIGURE 3:**
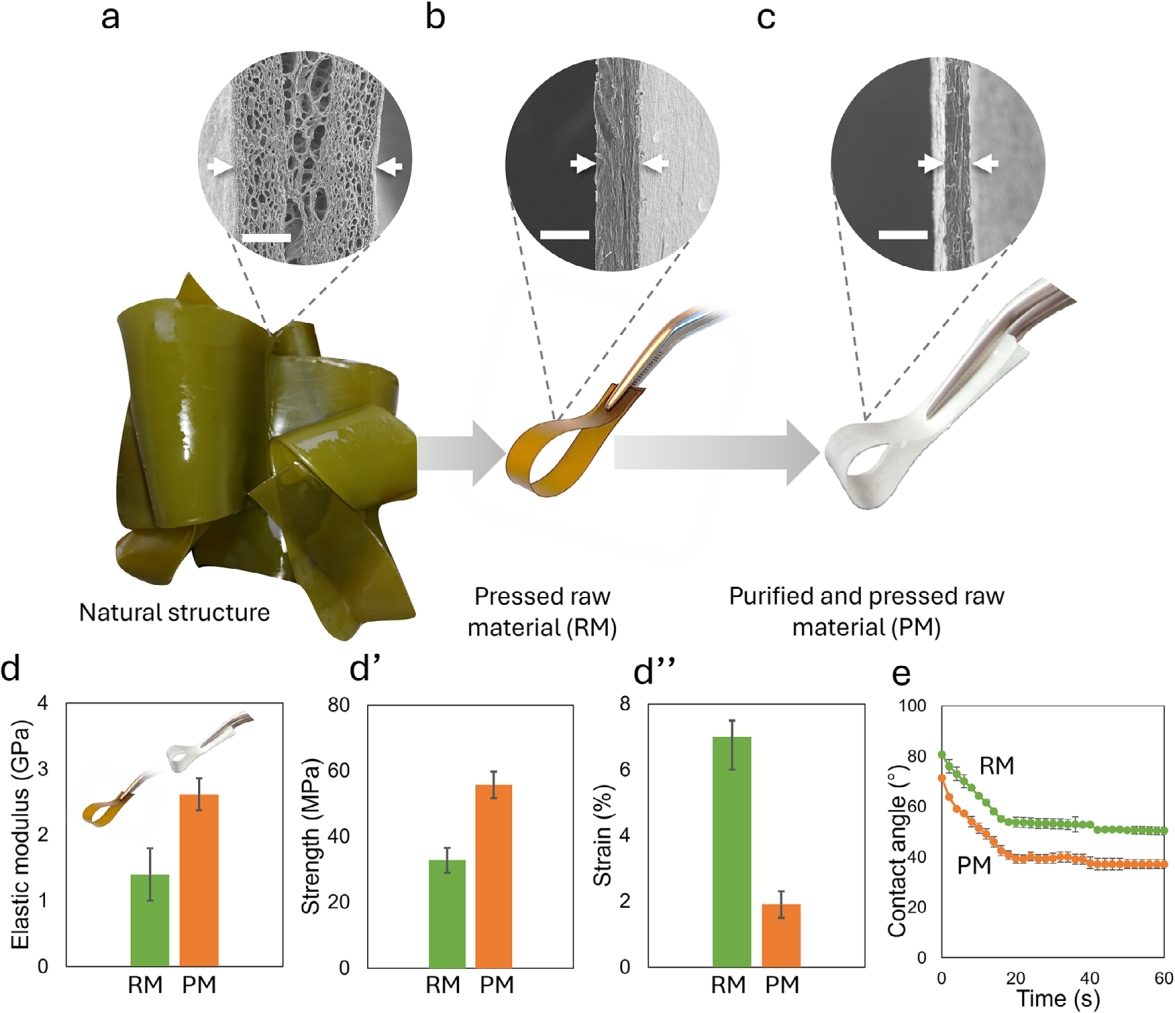
SEM images and photographs of cross‐sections for (a) raw blade natural structure (b) raw blade structure after pressing, (c) purified hydrogel structure after pressing—scalebar: 100 µm. Mechanical properties of the raw and purified (*n* = 10), pressed materials from tensile testing (d) elastic modulus, (d′) strength, and (d″) strain at break. (e) Contact angles as a function of time of raw and purified (*n* = 5), pressed materials, respectively.

To further investigate the influence of purification on functional properties, including mechanical behavior and hydrophilicity, the seaweed materials were hot‐pressed into dry films. Photographs and SEM images of the cross‐section of the raw blade, pressed raw blade, and purified pressed raw blade are shown in Figure 3a–c, respectively. Mechanical properties evaluated under tensile loading and contact angle measurements are presented in Figure 3d–d″,e, respectively.

As shown in Figure 3a–c, SEM analysis indicated that the porous structure of the raw seaweed was largely consolidated upon pressing. Furthermore, the bleaching process resulted in a thinner pressed material compared to the pressed raw structure. This difference in thickness was likely attributed to the removal of certain components during bleaching, which led to a looser internal structure that was more readily consolidated during pressing. Pressing was performed to enable tensile testing of the prepared materials. The purified and pressed networks exhibited a higher elastic modulus (Figure 3d) and tensile strength (Figure 3d′) than the pressed raw seaweed; however, the strain at break was more than three times higher for the raw seaweed structure (Figure 3d″). These results indicated that the purification process significantly altered the network structure and, consequently, the mechanical properties of the materials in the dry state (representative stress–strain curves provided in Figure S4). Sirviö et al. [19] reported the mechanical properties of CaCl_2_‐crosslinked films prepared by combining commercial sodium alginate and microfibrillated cellulose, in which the specific elastic modulus was more than three times higher, while the specific strength and strain at break were comparable to or slightly lower than those of the purified and pressed networks investigated in this study.

Surface wettability measurements for both networks displayed hydrophilic behavior (Figure 3e). The purification step resulted in overall lower contact angle values, indicating more pronounced hydrophilicity compared to the raw material. The purification process also appeared to alter the surface texture of the materials, as observed by SEM (Figure S5). This wettability behavior was likely influenced by differences in chemical composition and surface structure, which have previously been shown to affect wettability [20]. Sodium chlorite bleaching is known to remove pigments, phenolic compounds, proteins, and other non‐polysaccharide constituents from brown seaweed, while largely preserving the polysaccharide framework [21]. In a previous study, a total material yield of approximately 70% was reported after bleaching of brown seaweed. The bleached material contained approximately 23 wt.% cellulose and 46 wt.% alginate, whereas other residual components were not characterized [17]. In contrast, reported compositions for raw *Laminaria digitata* indicate alginate contents of approximately 25–30 wt.% and cellulose contents of 10–15 wt.% [22]. These differences suggest that bleaching substantially reduced non‐polysaccharide fractions, thereby increasing the relative abundance and surface exposure of hydrophilic polysaccharides. The enhanced wettability observed after purification can therefore be attributed to the increased availability of hydroxyl groups from cellulose and carboxylate groups from alginate, which promote hydrogen bonding with water and reduce the contact angle.

**FIGURE 4:**
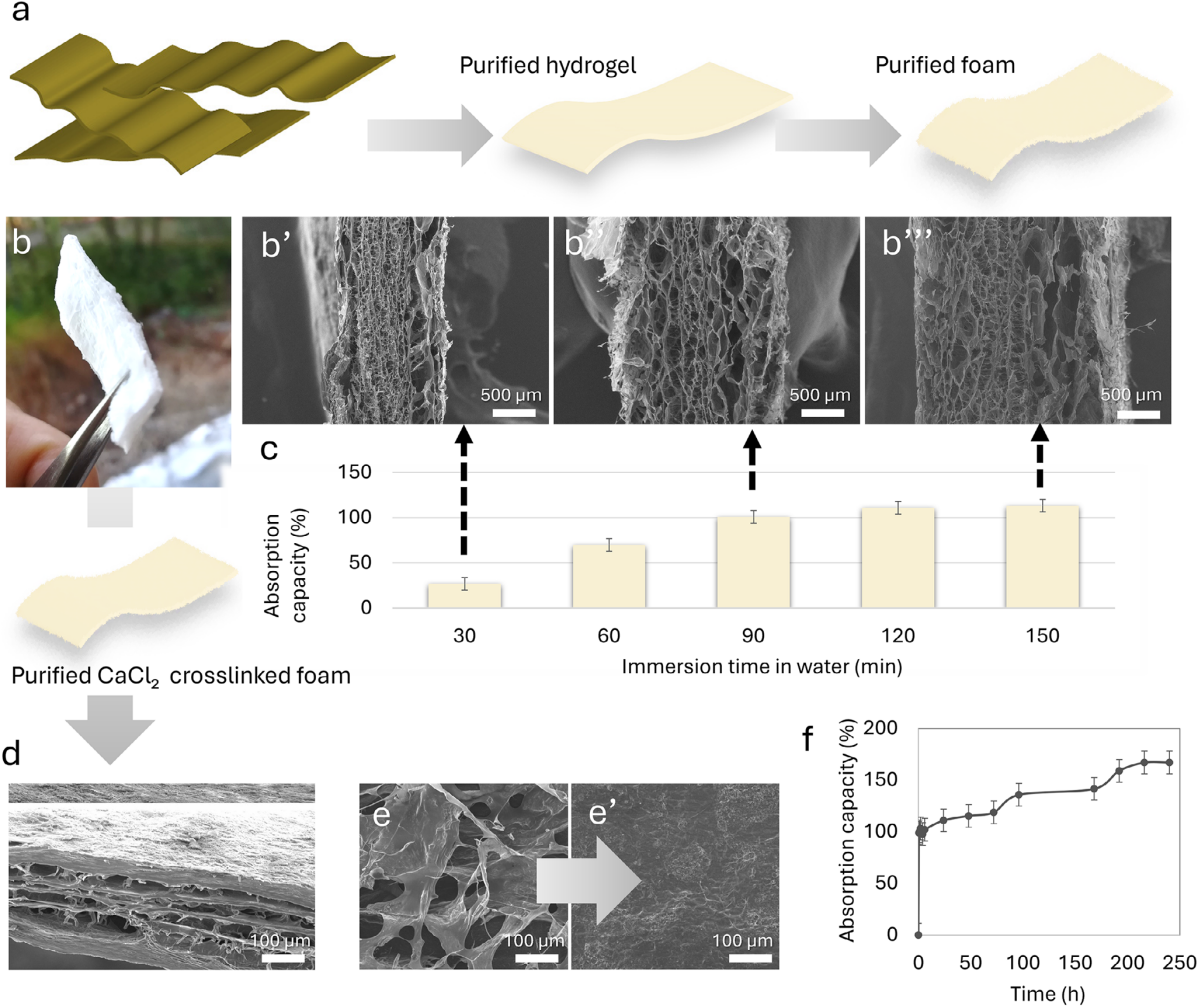
(a) Schematic overview of the raw blade structure, purified hydrogel structure, and foam structure after freeze‐drying of the purified hydrogel structure. (b) photograph of purified foam structure, and cross‐section SEM images of the purified foam structure after absorption in water for (b′) 30 min (b″) 90 min, and (b′″) 150 min, prior to freeze‐drying. (c) absorption capacity in water as a function of immersion time (*n* = 3). (d) SEM image of cross‐section of foam after CaCl_2_ crosslinking (e) surface before and (e′) surface after crosslinking. (f) absorption capacity in water after crosslinking foam structure (*n* = 3).

The purified seaweed structure was subsequently freeze‐dried to study its network structure using SEM and its absorption behavior through water submersion and capillarity‐driven uptake in aqueous red dye solution. In addition, crosslinking with CaCl_2_ was examined to achieve long‐term structural stability in water, and the results are presented in Figure 4 and Figure S6.

Upon water absorption over different time intervals, the non‐crosslinked foam exhibited an expanded network structure with larger pores, corresponding to longer absorption times and increased water uptake (Figure 4b–b’″). The foam reached a maximum absorption capacity of approximately 110% ± 14 (Figure 4c), after which structural integrity was lost. Udeni Gunathilake et al. [23] reported the development of superporous chitosan hydrogel reinforced with nanocellulose, achieving a maximum absorption capacity of approximately 400% for enhanced curcumin bioavailability. Key properties of wound dressing materials, including porosity, absorption capacity, hydrophilicity, and structural integrity, are commonly regulated using bioactive hydrogels to control fluid transport from the wound, enhance diffusion of encapsulated drugs, and facilitate the release of by‐products that support tissue regeneration [24]. Accordingly, water absorption behavior represents a critical characteristic of wound dressing materials, as effective absorption of excess wound exudate is essential for wound healing. Fluid absorption is partially governed by the capillary capacity of the material, which generally depends on pore size and pore density [24]. Preliminary testing of the purified seaweed foam structure was also conducted to evaluate potential capillary action, with photographs provided in Figure S4. The foam exhibited capillary uptake upon contact with liquid, suggesting that interconnected voids within the structure may have acted as channels for liquid transport. This observation further indicated that aspects of the layered architecture observed in the raw material were preserved to some extent, contributing to the capillary effect. However, suction capacity does not depend solely on porosity; accessible (open) porosity and pore tortuosity also influence capillary behavior [25]. Structural integrity is another critical requirement for biomedical materials; therefore, CaCl_2_ crosslinking was applied to enhance the hydrated stability of the foam over time. The effects of crosslinking on structure and absorption capacity were subsequently investigated. As shown in Figure 4d, crosslinking resulted in a denser network structure, as evidenced by SEM cross‐sectional images and by comparison of surface morphologies before and after crosslinking (Figure 4e,e′). Furthermore, the crosslinked foam exhibited a higher water absorption capacity and reached swelling equilibrium at approximately 160 ± 21% and remained structurally intact after 240 h of immersion, corresponding to 10 days in water (Figure 4f).

### 2.2 Reassembled Structure With Natural Composition

#### 2.2.1 3D‐Printed Hydrogel

**FIGURE 5:**
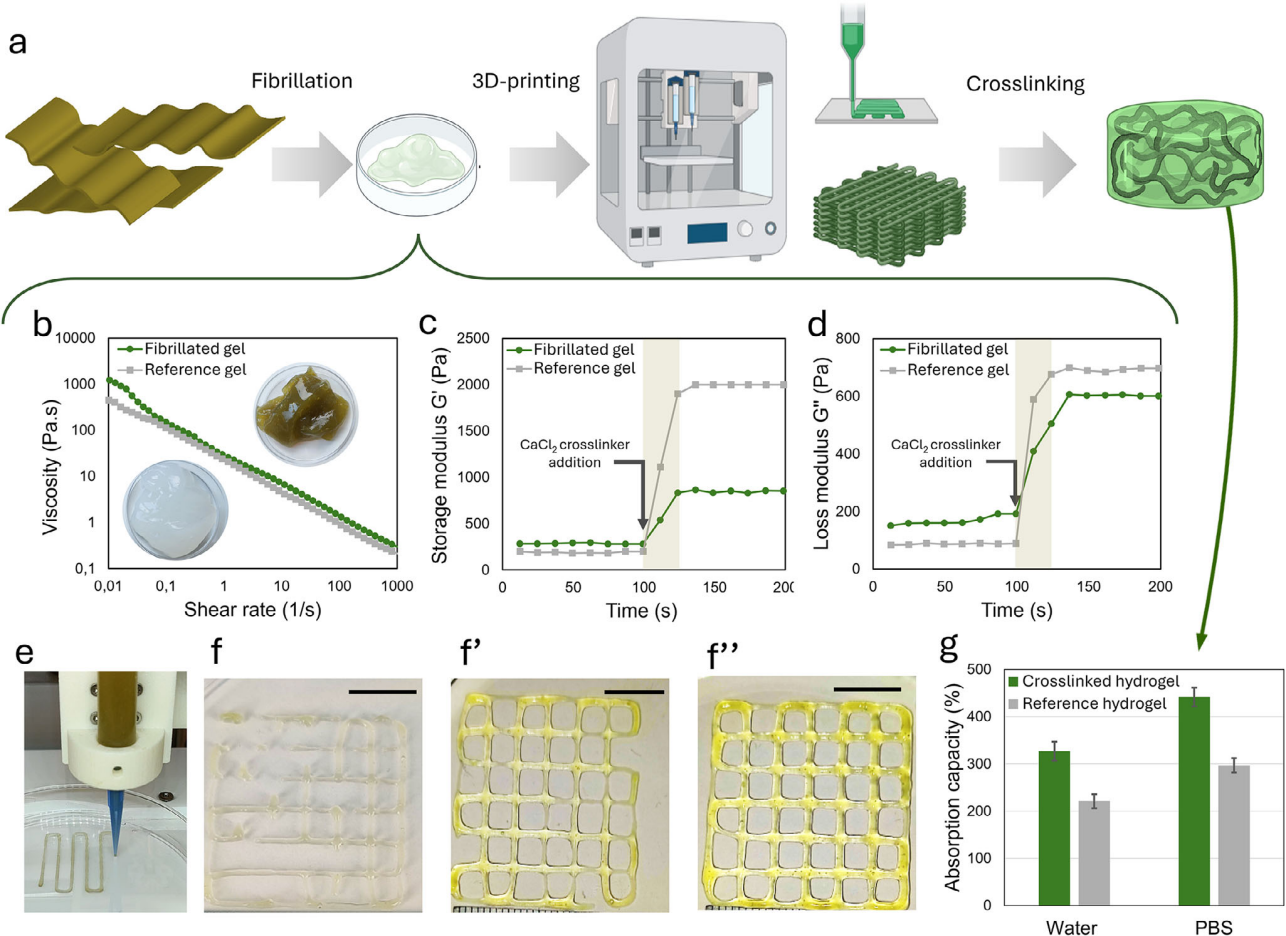
(a) Schematic process overview, (b) viscosity as a function of shear rate (*n* = 3), inset: photograph of fibrillated seaweed‐ and reference gel, (c) storage modulus‐ and (d) loss modulus as a function of time upon addition of crosslinker CaCl_2_ for fibrillated gel and reference gel (purified and fibrillated), respectively (*n* = 3). (e) Photograph of 3D‐printing of seaweed gel using (f) 7 kPa (f′) 9 kPa, and (f″) 10 kPa, of pressure, respectively, scalebar: 10 mm. (g) absorption capacity in water and PBS for cylindrical 3D‐printed hydrogel and purified hydrogel, respectively, after CaCl_2_ crosslinking (*n* = 3).

An additional approach involved fibrillation to generate nano‐ to microscale fibers, followed by reassembly into seaweed structures that were explored and optimized via 3D‐printing to form hydrogels (Figure 5). The fibrillated, 3D‐printed hydrogel structures were evaluated according to their ability to be crosslinked using CaCl_2_ as well as their structural integrity and absorption capacity in water and phosphate buffer saline (PBS). In addition, the seaweed was also bleached and fibrillated under the same conditions and used as a reference material for the evaluation according to rheology, 3D‐printability, shape fidelity, crosslink‐ability and absorption capacity and presented in Figure 5b–g and Figures S7–S8.

The seaweed was fibrillated at a concentration of 2 wt.%, and the measured viscosity increased from 987 ± 79 mPa.s before fibrillation to 4322 ± 121 mPa.s after fibrillation, indicating that the fibrillation process resulted in the formation of a stronger gel or network. The energy demand of the fibrillation process was 1.9 kWh/kg (dry weight). In comparison, the reference gel exhibited a viscosity of 1852 ± 119 mPa.s after fibrillation at a concentration of 2 wt.%.

The rheological properties of the fibrillated gels are critical for printability, with shear‐thinning behavior being particularly important for extrusion‐based 3D printing. Accordingly, the fibrillated gels were compared based on their viscosity as a function of shear rate. As shown in Figure 5b, the seaweed gel exhibited pronounced shear‐thinning behavior and a slightly higher initial viscosity than the reference gel. The high viscosity at low shear rates, combined with shear thinning at increasing shear rates, supported shape fidelity during printing; however, crosslinking of alginate was required to maintain structural integrity after printing. To achieve structural stability upon contact with liquids, the gels were crosslinked using CaCl_2_. The gelling behavior was examined by monitoring the storage modulus (G′) and loss modulus (G″) as functions of time during the addition of the crosslinker. As shown in Figure 5c,d, both moduli increased immediately following the addition of CaCl_2_ at approximately 100 s, with a more pronounced response observed for the reference gel. This behavior indicated a higher degree of crosslinking for the reference gel and resulted in increased stiffness or rigidity. Upon addition of crosslinking ions, rapid formation of intermolecular ionic bridges leads to the development of a three‐dimensional network, as reflected by the increase in storage modulus (G′) exceeding the loss modulus (G″), indicating the transition from a predominantly viscous to an elastic‐dominated response (Figure 5c). After approximately 20 s, a gradually linear response was observed (Figure 5c). The gelation process is governed by ionic crosslinking of alginate chains, primarily through divalent cation‐mediated coordination (e.g., Ca^2^^+^), forming junction zones consistent with the “egg‐box” model [26]. The fibrillated cellulose component further contributes to network formation by providing physical entanglement points and mechanical reinforcement, resulting in a hybrid ionic–physical network structure. The crosslinking kinetics can be interpreted from the time‐dependent evolution of G′ and G″, where the rate of modulus increase reflects the diffusion‐controlled ion–polymer interactions and progressive network stabilization. The plateau in G′ suggests completion of the primary crosslinking process and establishment of a mechanically stable gel network. The demonstrated 3D printability and crosslinking capability enabled the use of these gels in a wide range of applications, including those requiring defined geometries for wound dressings [27] and systems combining gels with cells for the 3D printing of living tissues and organs [28].

The 3D‐printability of the gels was evaluated based on extrudability and shape fidelity and optimized with respect to the applied printing pressure. As shown in Figure 5, printing at 7 kPa (Figure 5f) and 9 kPa (Figure 5f′) did not result in proper extrusion from the nozzle, as evidenced by the incomplete grid structures, compared with the highest applied pressure of 10 kPa, which produced the best shape fidelity (Figure 5f″). In contrast, printing the reference gel at 10 kPa led to ink spreading, and therefore this material could not be printed at pressures above 9 kPa (Figure S7a–a′″). The optimized 3D‐printed grid structures were further examined by optical microscopy to assess shape fidelity (Figure S8). Slightly rounded corners were observed; however, the printed structures exhibited comparable shape fidelity under the investigated conditions. The 3D‐printed structures remained structurally intact after crosslinking and exhibited water absorption capacities of approximately 300 ± 21% and 200 ± 16% for the hydrogel and purified reference hydrogel, respectively (Figure 5g). Reported literature values for water absorption capacities of alginate–nanocellulose mixtures range from approximately 50% [29] to 1400% [30], depending on mixture ratios and processing conditions. Absorption in PBS was slightly higher for both samples, with the hydrogel reaching equilibrium at approximately 450 ± 19% and the reference hydrogel at approximately 300 ± 17% (Figure 5g), consistent with previous observations for similar hydrogel systems [31].

#### 2.2.2 Freeze‐Dried Foam

**FIGURE 6:**
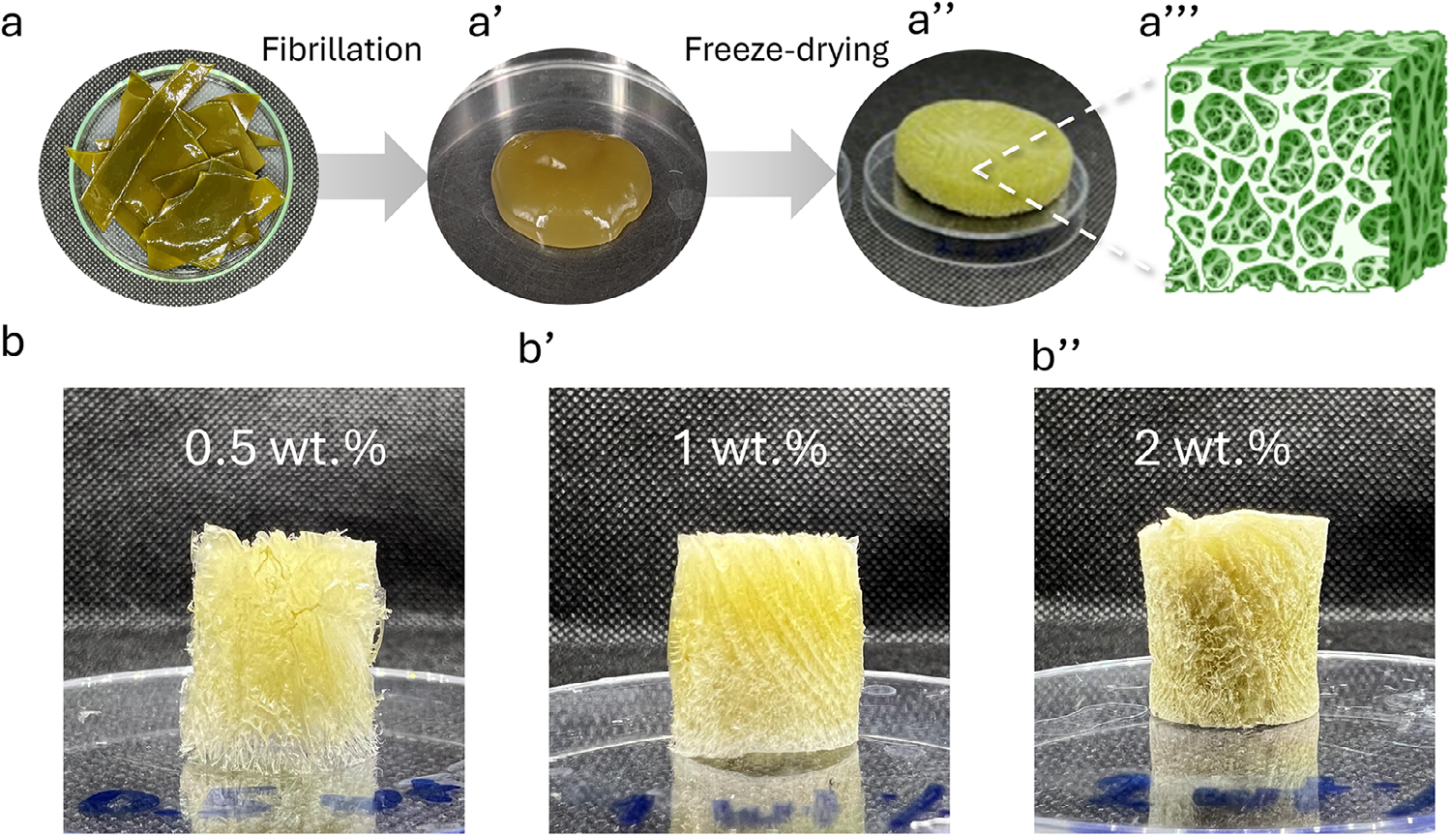
(a) Photographs of raw seaweed blade structure after (a′) fibrillation and (a″) freeze‐drying into porous foam structure, (a′″) schematic illustration of porous structure. Photographs of porous foam structures at different concentrations prior to freeze‐drying at (b) 0.5 wt.%, (b′) 1 wt.%, and (b″) 2 wt.%, respectively (dimension of the samples 14.5 mm diameter and ∼18 mm height).

In addition, the fibrillated seaweed was reassembled into porous structures via direct freeze‐drying, as shown in Figure 6. For this approach, the seaweed blades were fibrillated at a concentration of approximately 4 wt.% to enable higher material throughput and improve processing efficiency (Figure 6a,a’). The measured viscosity increased from 1850 ± 219 mPa.s before fibrillation to 8300 ± 233 mPa.s after fibrillation, which was approximately double the value obtained at a fibrillation concentration of 2 wt.%. Optical microscopy images acquired before and after fibrillation are provided in Figure S9, confirming a substantial reduction in structural size and the absence of larger intact seaweed fragments following fibrillation.

After fibrillation, the material was further diluted with water to obtain 0.5, 1, and 2 wt.% dispersions. These dispersions were subsequently cast into cylindrical molds and frozen at −23°C, followed by freeze‐drying for 24 h (Figure 6a″,a′″). Photographs of the resulting freeze‐dried samples at different concentrations are shown in Figure 6b–b″. Samples prepared at lower concentrations (0.5 and 1 wt.%) exhibited visually less dense structures and were therefore highly sensitive to manual handling, compared to the 2 wt.% sample, which could be handled without loss of structural integrity. The as‐prepared freeze‐dried materials were unstable in water; therefore, crosslinking with CaCl_2_ in an aqueous 3 wt.% solution was performed for the 2 wt.% samples. The crosslinked samples were subsequently evaluated in terms of porosity, absorption capacity (water and PBS), and shape stability under compressive loading. The physicochemical and mechanical properties of the freeze‐dried foam prepared at 2 wt.% are summarized in Table 1.

**TABLE 1:** Physicochemical and mechanical properties of the crosslinked freeze‐dried foam (2 wt.%) (*n* = 3).

Properties	Values
Percentage porosity (%)	93.3 ± 2.4
Water absorption (%)	2863.6 ± 276.4
PBS absorption (%)	3590.4 ± 215.1
Compressive stress (kPa)	10.3 ± 0.9

These materials exhibited a highly porous structure, with a calculated porosity of approximately 93%, which was comparable to values reported in literature for sodium alginate‐based foams (97%–90%) [32]. High porosity is advantageous for biomedical applications, as it facilitates nutrient diffusion and fluid exchange within the scaffold. The presence of micropores also contributes to the capillary‐driven fluid absorption, supporting the suitability of the material for exudate absorption and management. While porosity is known to influence absorption behavior, swelling in this system is additionally governed by polymer composition and crosslink density [33]. As shown in Table 1, the water absorption was approximately 2800%, which is also comparable to values reported for other seaweed‐based materials (2500%–3000%) [34], alginate‐based materials (2800%) [35], or commercial bacterial cellulose wound dressings (∼3100%) [36]. The foam was evaluated for PBS absorption, as its ionic strength is similar to that of wound exudate. PBS uptake provided insight into the material's ability to maintain a moist environment under physiologically relevant ionic conditions. Notably, the material showed substantially higher absorption in PBS, similar to the behavior observed for intact purified seaweed and 3D‐printed materials. This behavior suggested the involvement of ion‐exchange processes, in which carboxylate groups within the polymer matrix interacted with Na^+^ and K^+^ ions from PBS, partially replacing divalent calcium ions. This exchange likely enhanced polymer chain mobility and facilitated increased fluid penetration [37]. Partial disruption of ionic crosslinking may further loosen the polymer network, allowing greater absorption. In addition, the ionic strength of PBS likely reduced hydrogen bonding, resulting in a more expanded and hydrated network [31].

Compression testing was also performed to assess the mechanical stability of the foam structure. The maximum stress reached approximately 10 kPa at 50% strain (Table 1). The fibrillated foam exhibited excellent resilience (Video S1) and recovered its original height upon unloading, demonstrating shape‐memory behavior. The mechanical response is influenced by the material's microstructure and porosity; however, it is also dependent on network architecture and solid fraction. In highly porous systems, increased porosity generally results in greater compressibility and flexibility. The combined fluid absorption capacity and compressive integrity observed in this study indicate that foams derived from direct fibrillation exhibit a favorable balance between porosity‐driven absorption and network‐supported mechanical stability.

### 2.3 Outlook and Limitations of Seaweed Biomaterials

Nature has evolved hierarchical material architectures that achieve remarkable mechanical and functional performance through precise molecular organization. Brown seaweed exemplifies this design strategy, in which alginate and cellulose co‐assemble into a hydrated polymer network that combines flexibility, toughness, and effective water management. Translating these biological structure‐function relationships into engineered hydrogels represents a promising pathway toward multifunctional biomaterials. In this study, the native seaweed blade structure was purified to remove pigments and impurities, resulting in a visually intact blade structure with altered pore‐size distribution. The resulting hydrogel exhibited absorption capacity while maintaining structural integrity after ionic crosslinking. However, microscopy characterization and mechanical testing required drying of the samples, which limited insight into hydro‐responsiveness and structural behavior under fully hydrated conditions. Further investigation of wet‐state structure–property relationships is therefore required to fully understand material performance under physiologically relevant conditions. For fibrillated seaweed, the intrinsic composition enabled direct printability, shape fidelity, shear thinning behavior, and ionic crosslinking, without the need to tailor alginate‐to‐nanocellulose ratios, as commonly required for composite bioinks [28]. When reassembled into foam structures, fibrillated seaweed exhibited high absorption capacity, ease of crosslinking, high porosity, structural wet‐stability, and shape‐memory behavior, demonstrating potential for soft biomedical applications. The use of brown seaweed offers resource‐ and process‐efficiency advantages, including 100% material yield, an energy‐efficient fibrillation process, and elimination of hazardous chemicals. From a scalability perspective, upscalable techniques were applied to produce the gel structures, supporting their potential for large‐scale manufacturing. However, upscaling of the foam structures remains challenging due to the limitations of freeze‐drying. More broadly, natural biomaterials face scalability constraints related to maintaining consistent processing conditions, integrating unit operations, and establishing standardized quality control protocols, which can hinder commercial translation compared to fully synthetic materials [38]. In addition, inherent batch‐to‐batch variability—arising from differences in species, harvest location, seasonality, and extraction conditions—may lead to variations in polymer composition, molecular weight [39], gelation behavior, and mechanical performance [17], complicating reproducibility and regulatory approval for clinical applications.

### 2.4 Limitations and Cytocompatibility Considerations

Despite these promising functional properties, several limitations were identified that must be addressed for biomedical translation. First, the mechanical strength of the materials in the fully hydrated state remained limited, which may restrict their use in load‐bearing or long‐term applications. Second, the present study did not include an in vivo evaluation, and thus, biological performance, degradation behavior, and host response under physiological conditions remain to be established. A critical limitation identified was the cytotoxic potential of fibrillated seaweed gels at higher concentrations. Cytotoxicity testing was conducted using extract‐based evaluation according to ISO 10993‐5:2009 (Annex C), employing MTT assays with two extract concentrations (100% and 50%) (details provided in Table S1). This extract‐based approach represents a standardized and regulatory‐relevant preliminary screening method for assessing potential leachable‐induced cytotoxicity of medical device materials. The measured absorption values (reference subtracted) for the six replicate wells of the test item (100% extract), positive and negative control extracts (also 100% extract), respectively, are provided in Table S2, and the corresponding calculated viabilities are provided in Table S3 together with the cytotoxicity grading. The 100% extract of fibrillated seaweed gel (2 wt.%) exhibited a cell viability of 59.1 ± 3%, indicating cytotoxic potential according to ISO criteria (<70% viability). However, there was a notable difference between the negative control (102.5 ± 1.8%) and the fibrillated gel. Moreover, the positive control resulted in complete cell death (1.3 ± 0.1%). In contrast, the 50% extract yielded a viability of 73.4% and was classified as non‐cytotoxic, suggesting concentration‐dependent biological response and potential usability at reduced exposure levels. While the extract‐based MTT assay provides a standardized preliminary assessment, additional analyses, such as cell morphology imaging and longer‐term exposure studies, could provide deeper mechanistic insight into the observed concentration‐dependent effects and should be considered in future investigations. Furthermore, as the materials were processed under laboratory conditions and not under endotoxin‐controlled manufacturing environments, endotoxin quantification was not performed in this study. Endotoxin assessment and extended biological evaluation will be essential steps in future translational development. The observed cytotoxicity was likely associated with residual bioactive constituents inherent to brown seaweed, including proteins, endotoxins, and polyphenolic compounds [21]. Previous studies have reported that substantial amounts of residual proteins may remain even after extensive alginate purification, which can negatively affect cell viability [17]. In addition, phenolic compounds and endotoxins present in marine biomass may contribute to inflammatory or cytotoxic responses if not adequately controlled. Importantly, cytocompatibility is influenced by multiple factors, including raw material source, processing conditions, sterility, and residual chemicals. Previous toxicological assessments of purified fibrillated seaweed have demonstrated non‐cytotoxic behavior toward human fibroblasts [17], highlighting the strong dependence of biological response on processing history. Comparable indirect cytocompatibility values have been reported for alginate–nanocellulose hydrogels prepared from commercial components, with viabilities ranging from approximately 75% [40] to over 100% [28] depending on purification and formulation strategies. Notably, increased chemical modification or extensive purification does not inherently guarantee improved cytocompatibility. For example, TEMPO‐oxidized nanocellulose derived from purified wood pulp—widely regarded as a high‐performance biomedical material—has shown length‐dependent cytotoxic and inflammatory effects following extensive processing [41, 42]. These findings underscore that mitigation strategies should not rely solely on intensified chemical processing. Potential mitigation approaches for seaweed‐derived biomaterials may therefore include optimized washing protocols, controlled mild purification steps targeting specific cytotoxic constituents, dilution strategies, surface modification, or post‐processing treatments aimed at reducing bioactive impurities while preserving native structure. Collectively, the results indicated that the development of multifunctional hydrogel biomaterials based on seaweed's native architecture required a careful balance between minimal processing, functional performance, and cytocompatibility, rather than maximal purification alone

## 3 Conclusion

This study demonstrated that brown seaweed can be processed into hydrogel‐based biomaterials by leveraging its native structure–function relationships while limiting synthetic and chemical modification. Purification of the native blade structure enabled removal of pigments and impurities while preserving the hierarchical architecture, resulting in hydrogels with a broader pore‐size distribution and an absorption capacity of approximately 160%, together with maintained structural integrity for at least 10 days following CaCl_2_ crosslinking. For fibrillated seaweed, the intrinsic composition supported shear‐thinning behavior, printability, shape fidelity, and ionic crosslinking without the need for component ratio optimization. Compared with purified fibrillated systems, the non‐purified fibrillated seaweed exhibited higher viscosity and greater absorption in phosphate‐buffered saline (approximately 450% vs. 300%), indicating a strong influence of retained native constituents on functional behavior. When reassembled into freeze‐dried foam structures, fibrillated seaweed achieved high porosity (∼93%) and absorption capacities of approximately 2900% in water and 3600% in PBS, alongside structural stability, crosslinkability, and shape‐memory response under compression. Cytocompatibility assessment revealed concentration‐dependent biological response, with fibrillated seaweed gel extracts showing reduced viability at full concentration (59.1 ± 3%) but non‐cytotoxic behavior at 50% extract (73.4%). These findings highlighted the importance of balancing minimal processing with biological performance. Overall, the results underscore the potential of utilizing seaweed's native architecture and composition to develop resource‐efficient hydrogel biomaterials with tunable absorption and structural properties, while identifying cytocompatibility and processing trade‐offs that must be addressed for future biomedical applications.

## 4 Experimental Section

### 4.1 Materials

Brown seaweed (*L. digitata*) was kindly provided by The Northern Company Co. (Træna, Norway) and used as the raw material in this study. The fresh samples were stored in closed bags in the freezer before use. Sodium chlorite (NaClO_2_), sodium hydroxide (pure pellets, NaOH), and acetic acid (96% CH_3_COOH) were purchased from Merck KGaA, Darmstadt, Germany. For cross‐linking, calcium chloride (CaCl_2_) was used from Sigma–Aldrich Sweden AB, Stockholm, Sweden. PBS was also purchased from Sigma–Aldrich Sweden AB, Stockholm, Sweden. All chemicals were used as received. Deionized water was used for all experiments.

### 4.2 Purification of Seaweed Structure

The blades of the seaweed were left at room temperature (22 ± 1°C) for about 24 h to defrost. The raw blade structure was subsequently purified by bleaching with NaClO_2_ (1.7%) in an acetate buffer (pH 4.5) at 80°C for 30 min, resulting in an intact hydrogel structure. Another batch was prepared to be used as a reference material for the fibrillated seaweed, where the seaweed was cut into smaller pieces and bleached under the same conditions. After purification, all colors were removed from both batches prepared, and the material was washed until a neutral pH was reached. The crosslinking of the samples was performed by immersing the samples in a 90 mM aqueous solution of CaCl_2_ for 2 h. The crosslinked samples were subsequently rinsed with deionized water until excess of crosslinker was removed. The purified seaweed structure was frozen in its intact hydrogel form after purification and after crosslinking and was subsequently freeze‐dried for further characterization.

### 4.3 Fibrillation of Seaweed

The materials (at a concentration of 2 and 4 wt.%) were fibrillated using an MKZA6‐3 ultrafine friction grinder, Masuko Sangyo Co., Ltd. (Kawaguchi, Japan), with coarse silica carbide (SiC) grinding stones. The structures were intact after cutting, hence the first pass through the Masuko grinder was performed with an open gap (20 µm) to pre‐disperse the materials, given that no other dispersing equipment was used. The fibrillation was conducted in contact mode, with the gap between the two discs gradually adjusted to −90 µm, at 1500 rpm. The fibrillated gel was stored in the refrigerator until further assembly into hydrogel and foam structures. The purified reference gel was fibrillated using the same fibrillation settings. The energy consumption of the fibrillation process was established by the direct measurement of the power with an energy analyzer, EM24 DIN, Carlo Gavazzi (Belluno, Italy) and from the monitored processing time. The energy demand was calculated from the product of the power and the time, and the energy consumption is expressed as kilowatt‐hour per kilogram of dry weight material. The first sample was collected after the first pass through the Masuko grinder with an open gap. Samples were subsequently collected at regular intervals to assess the degree of fibrillation. The process was finalized when a plateau was reached in viscosity, and no larger structures could be observed by a microscope.

### 4.4 3D Printing of Fibrillated Seaweed Into Hydrogels

Seaweed gel and reference gel were evaluated for printability and shape fidelity at 2 wt.% and without additional processing steps after the fibrillation process. Cylindrical discs and grid structures were 3D printed using an INKREDIBLE 3D bioprinter, CELLINK AB, (Gothenburg, Sweden), which is a pneumatic‐based extrusion bioprinter. Different designs were made using Tinkercad (Autodesk) software. A one‐layered zigzag pattern with long line 30 mm and short line 5 mm, a ten‐layered hollow cylinder with a diameter 20 mm, and a two‐layered grid with dimensions 30 × 30 mm^2^ was made using the CAD software 123D Design (Autodesk) and subsequently imported to Cellink Heartware (Cellink Inc.) software for slicing prior to 3D‐printing. A nozzle diameter of 0.4 mm was used at a pressure varying between 5 and 10 kPa and a dosing distance of 0.05 mm. The printing parameters were determined from the visual printing resolution of grid structures (15 × 15 mm^2^, three layers). The two ink formulations were 3D printed directly onto a glass Petri dish, then cross‐linked in a bath of a 90 mM aqueous solution of calcium chloride for 30 min directly on the Petri dish, and finally washed with deionized water.

### 4.5 Freeze‐Drying Into Porous Foams

The fibrillated (4 wt.%) seaweed gel at a concentration varying between 0.5 and 2 wt.% was reassembled into porous foam structures in a cylindrical mold (dimensions 15.6 mm diameter and 20 mm height), freezing overnight at −23°C, followed by freeze‐drying using freeze‐dryer Alpha 1–4 LD plus LSC (Labex of Scandinavia AB, Helsingborg, Sweden) with Lyo Cube front loader at 1 mbar pressure for 24 h. Dried samples were stored at room temperature until further characterization. The crosslinking of the samples was performed by immersing the samples in a 3 wt.% aqueous solution of CaCl_2_ for 2 h. The crosslinked samples were taken out carefully and rinsed with deionized water until excess of crosslinker was removed.

### 4.6 Optical Microscopy

An optical microscope, Nikon Eclipse LV100N POL (Kanagawa, Japan), and the imaging software NIS‐Elements D 4.30 were used to study how the fibrillation process affected the material structure and its micrometer‐scale size. Polarized optical microscopy (POM) reference images with a polarization filter were also captured. Microscopy was also used to assess the shape fidelity after 3D‐ printing of grid structures.

### 4.7 Viscosity

Measurements were performed during the fibrillation process to evaluate the degree of fibrillation, and the process was stopped once a viscosity plateau was reached. A Vibro Viscometer SV‐10 from A&D Company, Ltd. (Tokyo, Japan) was used at a constant shear rate and with periodical circulation of the sensor plates from zero to peak (sine‐wave vibration) at a frequency of 30 Hz. Because the temperature increased during the fibrillation owing to the compression and abrasive shearing forces, the viscosity measurements were repeated at a stabilized temperature of 22.1 ± 1.1°C to confirm that a viscosity plateau had been reached during the process. The presented values are an average of three measurements for each sample.

### 4.8 Scanning Electron Microscopy (SEM)

SEM system (JSM‐IT300LV, Tokyo, Japan) at an acceleration voltage of 15 kV was used to study the microstructure of the different seaweed structures, including the natural seaweed structure before and after purification and after pressing, freeze‐drying, and crosslinking to study the processing effects on the microstructure. All samples were coated using a sputter–coater machine (Leica EM ACE200, Austria) with a gold target to prevent electron charging. The coating was performed in a vacuum of approximately 6 × 10^−5^ mbar under a current of 100 mA for 20 s to obtain a coating thickness of 10 nm.

### 4.9 X‐Ray Microtomography (XRT)

The internal 3D structure of the raw seaweed blade and the purified seaweed blade was reconstructed after freeze‐drying. Samples of approximate size 64 mm^3^ were scanned using a Zeiss Xradia 510 Versa (Carl Zeiss, Pleasanton, CA, USA) with a 20× objective, with a field of view of 0.56 mm and voxel size of 0.56 µm. The scanned volume of interest was positioned at the exact center of each sample to visualize the internal structure using an X‐ray tube voltage of 50 kV, an output power of 4 W without X‐ray filters. A total of 2401 projections were acquired with an exposure time of 6 s, which resulted in a total scan time of 6 h. The reconstruction was carried out using filtered back‐projection with Zeiss Scout‐and‐Scan Reconstructor software (version 11.1) and the 3D visualization and analysis of the seaweed architecture were obtained using Dragonfly Pro Software (ORS).

### 4.10 Mechanical Properties

The natural and purified hydrogel structure was pressed using a hot‐press to enable testing of its mechanical properties. The pressed structures were tested using conventional tensile testing to provide an indication of how the purification step affected the natural structure. Prior to mechanical testing, the density was calculated using a gravimetric method: an analytic balance was used to determine the weight, and a micrometer gauge was used to measure the thickness based on an average of 10 different measurements per material. A tensile testing system (AG X; Shimadzu, Japan) equipped with a 1 kN load cell was used at an extension rate of 2 mm/min and with a gauge length of 20 mm. All samples were conditioned at 50 ± 2.0% relative humidity at 22.1 ± 1.0°C for at least 48 h. The results were averages of at least 10 sets of measurements for each material. Compression test of fibrillated freeze‐dried samples was tested in wet conditions (samples were soaked in water for 24 h prior to test) using CT3 1000 Texture Analyzer (Cromocol Scandinavia AB, Borås, Sweden) in a single compression cycle compression mode. The deformation was set at 50%, trigger at 0.005 N, and the speed was set at 10 mm/s throughout all the experiments. Three independent replicates were analyzed for compression loading.

### 4.11 Percentage Porosity

Percentage porosity was calculated using the solvent displacement method with some modifications [19, 43]. The sample with known mass and dimensions was immersed in ethanol for 48 h. The mass of ethanol absorbed samples was noted down for further calculation. The following formula was used to calculate percentage porosity:

(1) $$Percentage\;porosity\;\left(\%\right)=\frac{W_{t}-W_{d}}{\rho V_{d}}\;\times 100$$

here, *W*_d_, *W*_t_, *V*_d,_ and *ρ* are the initial weight of the sample, weight of the sample at saturation (in EtOH), known volume of the sample at start of the study, and density of EtOH at 20°C (0.7892 g/mL), respectively. Three independent replicates were analyzed.

### 4.12 Water Contact Angle

The wettability of the natural and purified hydrogel pressed structures was characterized using water contact angle measurements with an EASYDROP measuring system, drop shape analysis control (DSA1), and evaluation software (Krüss GmbH, Hamburg, Germany). A 4 µL water drop was placed onto the samples, and the contact angle was calculated using the sessile drop technique. The reported values were the average of five measurements for each sample.

### 4.13 Absorption Capacity

The different seaweed structures were evaluated according to their water absorption capacity by immersion in water at room temperature, and their weight was monitored over time. Excess water was removed by gently tapping the samples on dry tissue paper. Initially, the purified seaweed structure was evaluated after freeze‐drying to study the effect of absorption time and crosslinking. The 3D‐printed nanofiber seaweed hydrogels were also evaluated after CaCl_2_ crosslinking to study the effect of bleaching. Finally, the reassembled seaweed microfiber foam structure after freeze‐drying was evaluated. The absorption was calculated as follows:

(2) $$Absorption\;capacity\;\left(\%\right)=\frac{W_{t}-W_{d}}{W_{d}}\times 100$$

where *W*_d_ denotes the initial weight of the dried sample and *W*_t_ is the weight at time *t* after immersing the samples in water. PBS absorption was also studied for foams and 3d printed structures in the similar manner and was calculated using the same formula. Experiments were conducted using three replicates.

### 4.14 Capillary Effect

To establish the capillary effects in the purified seaweed structure after freeze‐drying, a capillary rise experiment was carried out using water colored with Congo red dye. The liquid was placed in a container, and broad strips (10 mm width to avoid edge effects) of the samples were suspended such that the ends were just in contact with the liquid.

### 4.15 Rheology

Rheological analysis was carried out on the inks, using a Discovery HR‐20 rheometer from TA Instruments (Elstree, UK), maintained at room temperature (23 ± 1.0°C). For these tests, a parallel‐plate configuration (20 mm) was utilized to measure shear viscosity across a range of shear rates from 0.1 to 1000/s. Additionally, the changes in moduli during the cross‐linking of the inks were assessed using a plate‐plate configuration with a geometry gap of 550 µm. Oscillation frequency measurements were conducted at a strain of 0.1% and a frequency of 1 Hz over a duration of 10 min, with oscillation amplitude sweeps employed to define the linear viscoelastic region. Crucially, 120 s after commencing these measurements, 3.5 mL of crosslinker solution was added to the inks. Triplicate samples were used for 90 mM calcium chloride. The addition triggered gelation, allowing for simultaneous measurement of the storage and loss moduli, thereby providing deeper insights into the gelation dynamics and structural integrity of the inks as a result of crosslinking.

### 4.16 Cytotoxicity

The cytotoxic potential of fibrillated seaweed gel was determined using extraction of the test item and cytotoxicity testing according to ISO 10993‐5:2009 Annex C. Extracts from test item, positive and negative controls as well as blanks (extraction vehicle not containing the test item but subjected to conditions identical to those to which the test item was subjected to during extraction) were added to a subconfluent monolayer of L929 mouse fibroblast cells and incubated for 24 h at 37 ± 1°C in 5 ± 1% CO_2_. Each extract solution was added to 6 replicate wells, containing a subconfluent monolayer of cells. Blanks were also placed in 6 wells on each side of the 96 well plate to confirm that no systematic cell seeding errors occurred, as well as to serve as a 100% measure of cell viability. After incubation, the extracts were removed and MTT solution was added to the cells which were incubated for an additional 2 h at 37 ± 1°C in 5 ± 1% CO_2_. Following incubation, the MTT solution was removed, 2‐propanol was added, and the plates were shaken rapidly. Finally, the absorbance was measured at 570 nm (reference wavelength 650 nm), and the viability of cells was calculated.

## Conflicts of Interest

The authors declare no conflicts of interest.

## Supporting information




**Supporting File**: mabi70182‐sup‐0001‐SuppMat.pdf.

**Supporting File**: mabi70182‐sup‐0002‐VideoS1.mp4.

## Data Availability

The data that support the findings of this study are available from the corresponding author upon reasonable request.
