# Supplementary material for: Integrating Biological Architecture and Biomaterial Function: Exploring the Native Hydrogel Structure of Brown Seaweed
Source: Macromol Biosci. 2026 Apr 7;26(4):e00622. doi: 10.1002/mabi.202500622 (PMC13054773; doi:10.1002/mabi.202500622)
Supplement: Supplementary file 1 — Supporting File: mabi70182‐sup‐0001‐SuppMat.pdf. [file MABI-26-e00622-s002.pdf]

# Supporting Information for

## Integrating Biological Architecture and Biomaterial Function: Exploring the Native Hydrogel Structure of Brown Seaweed

*Linn Berglund* and Richa Sharma*

Division of Materials Science, Department of Engineering Sciences and Mathematics, Luleå University of Technology, SE-971 87 Luleå, Sweden

E-mail: linn.berglund@ltu.se

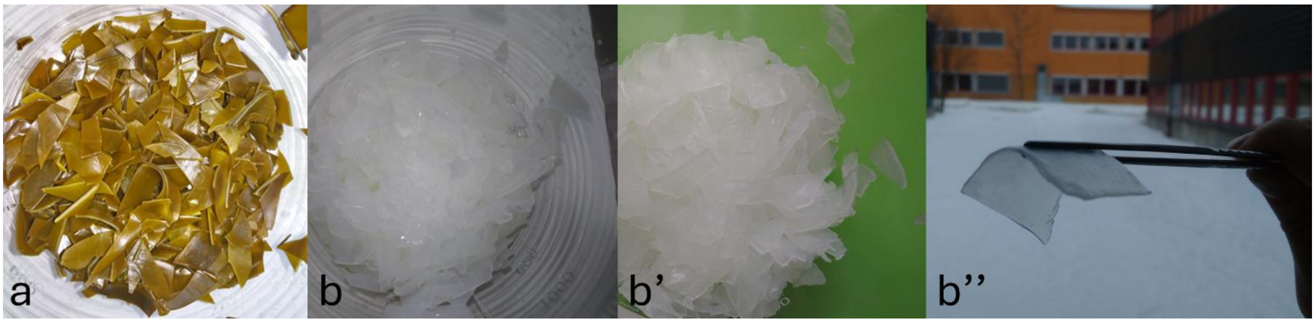


**Figure S1.** Photographs of seaweed blades (a) before and (b, b’, b’’) after purification.

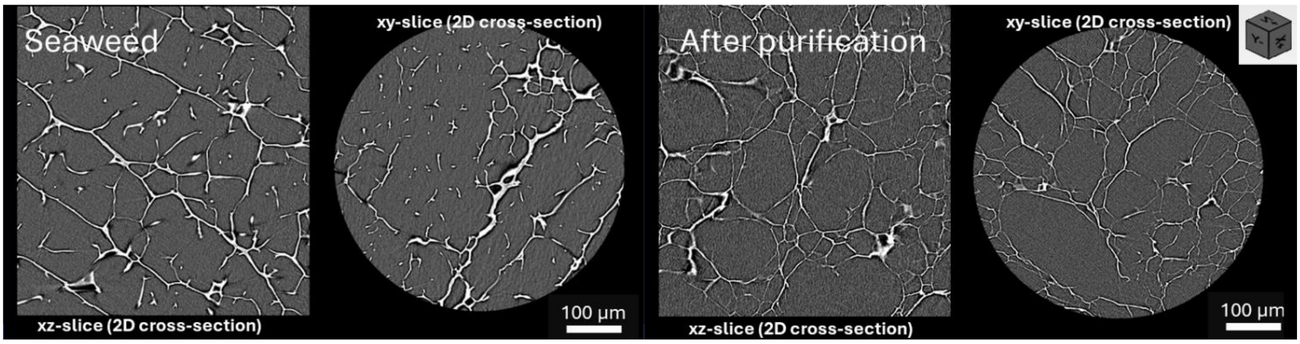


**Figure S2.** XRT 2D cross-section from 3D scanning of the seaweed raw blade structure and the purified seaweed blade structure.

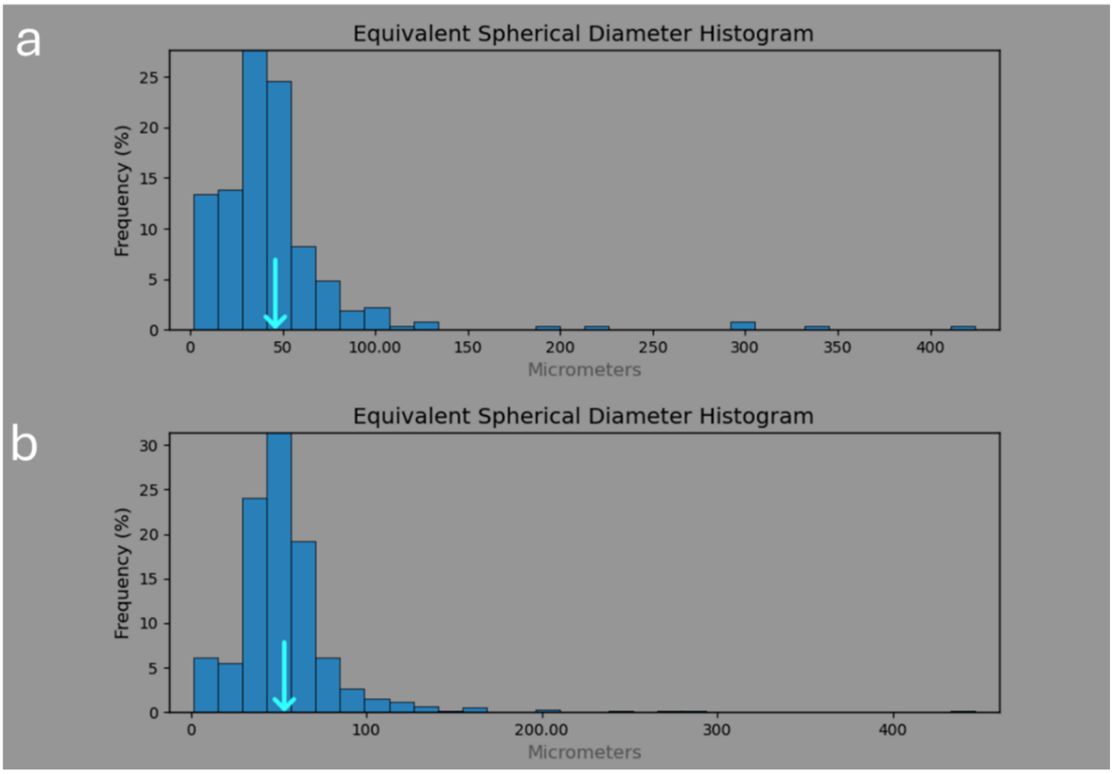


**Figure S3.** Pore-size distribution from segmentation of XRT reconstructions of (a) raw blade structure and (b) purified blade structure.

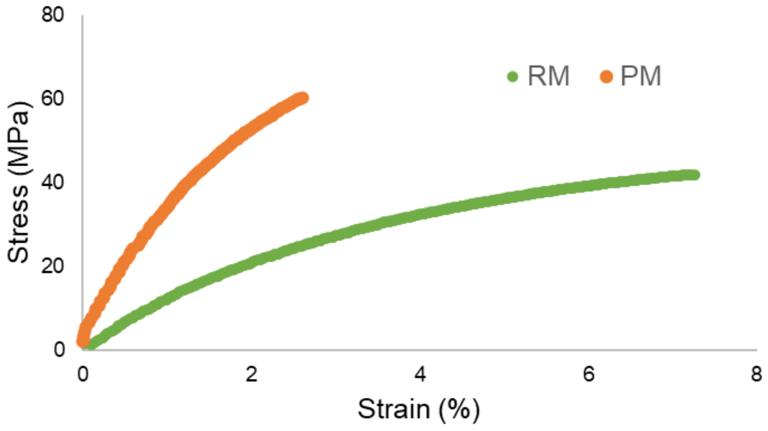


**Figure S4.** Representative stress-strain curves from tensile testing of the seaweed raw blade structure (RM) and the purified seaweed blade structure (PM), after pressing.

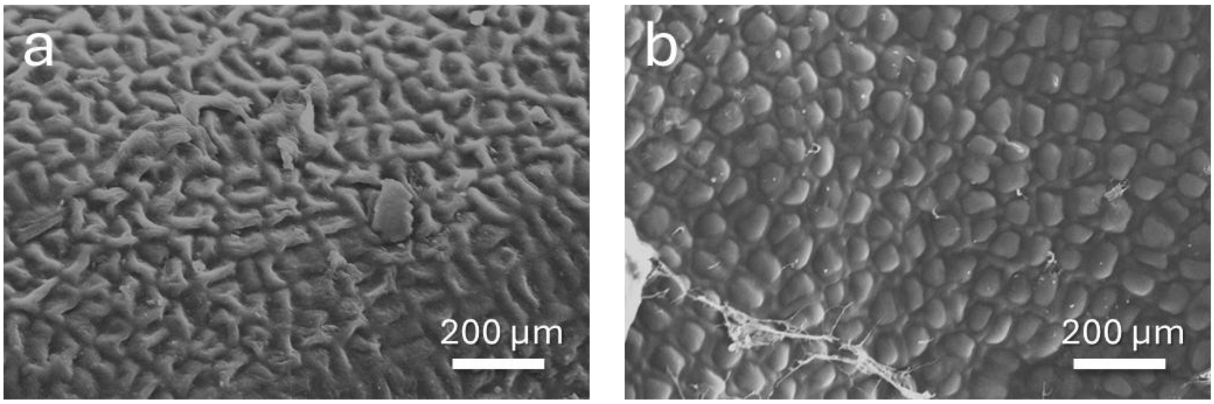


**Figure S5.** SEM images of seaweed blade surface (a) before and (b) after purification.

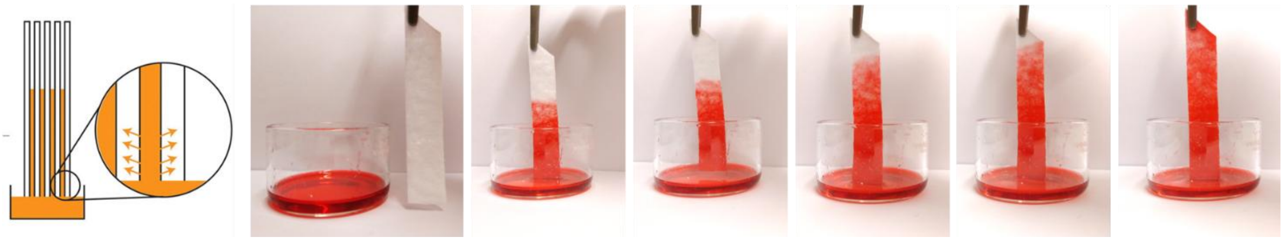

**Figure S6.** Capillary rise measurements in contact with water with congo-red dye.

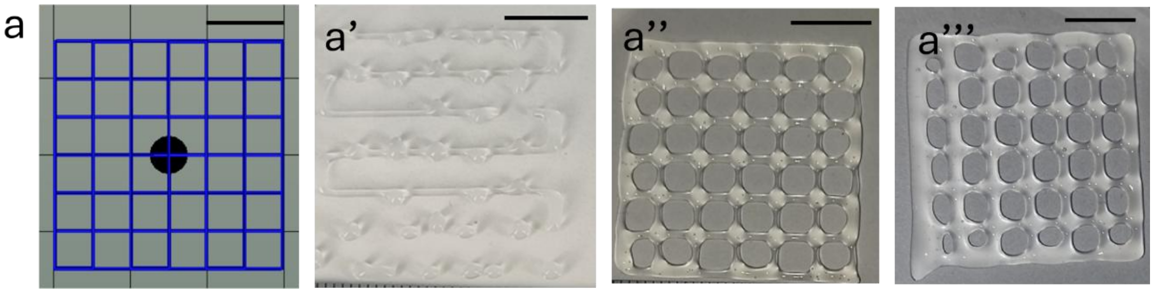


**Figure S7.** (a) CAD grid structure for 3D-printing and photographs of 3D-printed nanofiber seaweed gel using (a’) 7 kPa (a’’) 9 kPa, and (a’’’) 10 kPa, of pressure, respectively.

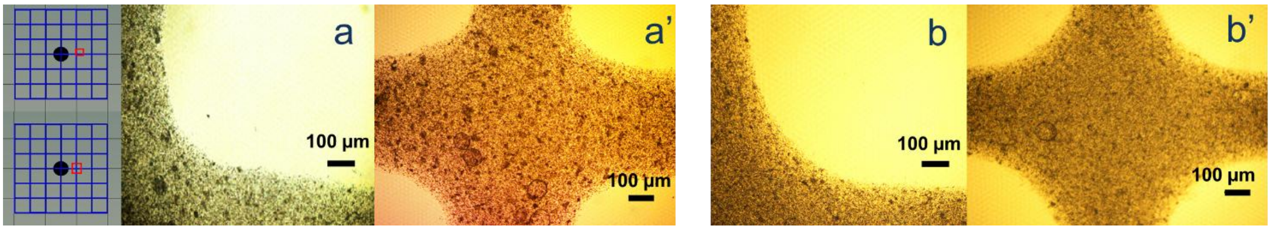


**Figure S8.** Indication of visualized area on 3D-printed grid structure for seaweed hydrogel (a) corner and (a’) cross-section, and purified seaweed hydrogel (b) corner and (b’) cross-section, respectively.

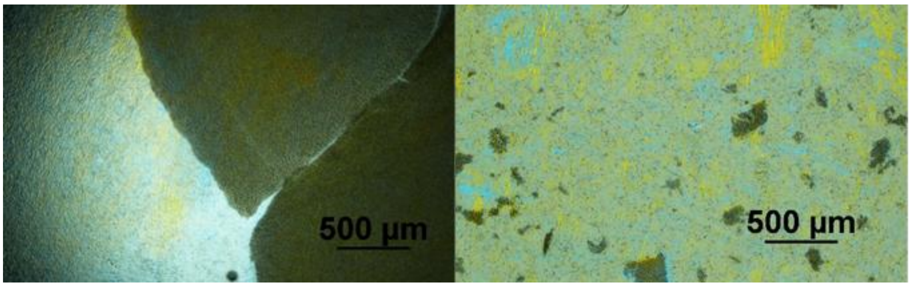


**Figure S9.** Optical microscope images of seaweed blades before and after fibrillation.

**Table S1**. Extraction details.

| | |
|---|---|
| Extraction ratio | 0.2 g/ml |
| Extraction volume | 6.22 ml |
| Extracted area or mass | 1.2445 g |
| Extraction vehicle | Eagle’s Minimum essential medium 1X with non-essential amino acids and sodium pyruvate, supplemented with 5% (v/v) Fetal Bovine Serum, 4 mM L-glutamine, 100 IU/ml penicillin and 100 µg/ml streptomycin |
| Extraction duration | 24 ± 2 hours |
| Extraction conditions | 37 ±1 °C with agitation (30 rpm) |

**Table S2.** Measured and averaged absorption values for the six replicates of the test item, positive and negative control.

| RISE identity | Absorption (570 nm -650 nm) | | | Average | Standard deviation |
|---|---|---|---|---|---|
| 9P06940:2 | 0.387 | 0.417 | 0.432 | 0.424 | 0.021 |
| | 0.449 | 0.436 | 0.423 | | |
| Positive control | 0.009 | 0.009 | 0.011 | 0.010 | 0.001 |
| | 0.009 | 0.008 | 0.011 | | |
| Negative control | 0.727 | 0.749 | 0.746 | 0.735 | 0.013 |
| | 0.717 | 0.745 | 0.728 | | |

**Table S3.** Calculated viabilities and cytotoxicity grading according to ISO10993-5:2009 for the six replicates of the test item, positive and negative control.

| RISE identity | Viability (%) | | | Average | Standard deviation | Cytotoxicity grading |
|---|---|---|---|---|---|---|
| 9P06940:2 | 53.9 | 58.1 | 60.3 | 59.1 | 3.0 | Cytotoxic |
| | 62.6 | 60.7 | 58.9 | | | |
| Positive control | 1.3 | 1.2 | 1.5 | 1.3 | 0.1 | Cytotoxic |
| | 1.3 | 1.2 | 1.5 | | | |
| Negative control | 101.3 | 104.4 | 104.0 | 102.5 | 1.8 | Not Cytotoxic |
| | 100.0 | 103.9 | 101.5 | | | |

* The measured viability of test item extract 50% was 73.4%.
